# Discriminating between native and phosphorylated tau proteins using fluid‐lipid bilayer‐coated nanopores

**DOI:** 10.1002/alz.086347

**Published:** 2025-01-09

**Authors:** Saurabh Awasthi, Michael Mayer

**Affiliations:** ^1^ National Institute of Pharmaceutical Education and Research, Raebareli (NIPER‐R), Lucknow, Uttar Pradesh India; ^2^ University of Fribourg, Adolphe Merkle Institute, Fribourg Switzerland

## Abstract

**Background:**

Tau protein phosphorylation and aggregation are the pathological hallmarks of Alzheimer's disease (AD) and other tauopathies. Multiple phosphorylation sites in Tau protein at serine (S), threonine (T), and tyrosine result in high heterogeneity and enhanced aggregation kinetics.

**Method:**

Here, we used nanopores coated with a fluid lipid bilayer to characterize native and hyperphosphorylated Tau proteins on a single‐molecule level.

**Result:**

Single‐molecule level analysis determines the size, shape, net charge, and dipole moment of individual tau protein monomers. The approach makes it possible to differentiate native and hyperphosphorylated Tau proteins based on their net charge. Moreover, single‐particle size analysis revealed the spontaneous formation of low‐n oligomers such as dimer, trimer, tetramer and pentamers; no other molecules were needed to initiate aggregation to these oligomers.

**Conclusion:**

The nanopore‐based approach enables discrimination of native and hyperphosphorylated Tau proteins based on their net charge. Furthermore, single‐particle size approximation is helpful for detecting early‐stage aggregation of Tau and characterization of various oligomer species.

